# A Rare Case of Isolated Prostate Recurrence From Testicular Seminoma

**DOI:** 10.7759/cureus.48385

**Published:** 2023-11-06

**Authors:** Joana Albuquerque, Teresa Timóteo, Ana Pestana, José Passos-Coelho

**Affiliations:** 1 Oncology, Hospital da Luz Lisboa, Lisboa, PRT; 2 Pathology, Hospital da Luz Lisboa, Lisboa, PRT

**Keywords:** prostate metastases, bep, combined chemotherapy, metastatic seminoma, testicular germ cell tumors

## Abstract

We report the case of a 32-year-old male diagnosed with a left-sided testicular seminoma treated with radical inguinal orchiectomy and staged as pT1bN0M0S0 (*rete testis *invasion) - stage IA. Adjuvant treatment options were discussed, and active surveillance was chosen. Two years later, he presented with urinary retention alternating with pollakiuria, a feeling of incomplete bladder emptying, dyspareunia, and anejaculation. A rectal examination documented an enlarged, nodular, painful prostate. Blood and urine analyses, including serum tumor markers, were unremarkable. Pelvic magnetic resonance (MR) documented a central, nodular, solid, hypermetabolic, prostatic tumor with a size of 40x50x25 mm, invasion of the right seminal vesicle, right anterolateral wall of the rectum, and postero-inferior bladder wall, and an absent lymph node and visceral disease. A transrectal ultrasound-guided (TRUS) biopsy documented prostatic metastasis of the seminoma. The patient was treated with four cycles of bleomycin, etoposide, and cisplatin (BEP) chemotherapy (ChT) with a complete (clinical, radiologic, metabolic, and pathological) response. After five years of follow-up, he remains asymptomatic without a recurrence of the disease.

## Introduction

Testicular germ cell tumors (GCTs) are the most frequent solid tumors in young men between 25 and 35 years old, accounting for 1-2% of all types of cancer in men. The majority are stage I seminomas, which are associated with a very good prognosis regardless of the treatment approach [1,2]. Classically, testicular seminomas spread first to the retroperitoneal lymph nodes and, eventually, to the lungs, liver, and brain. Metastasis of the prostate is a very unusual metastatic site [1-3]. We report a rare case of isolated prostatic metastasis from a testicular seminoma.

## Case presentation

**Figure 1:**
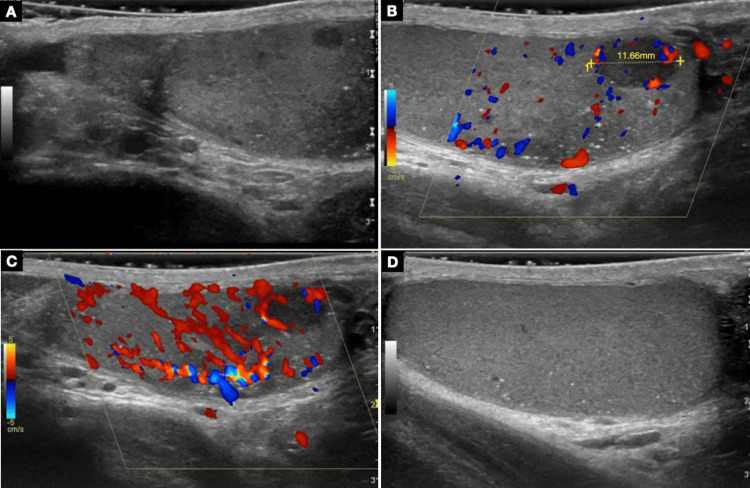
Scrotal ultrasonography of the left testis showing a markedly heterogeneous structure, with hypo-echoic areas, (A) microlithiasis, and (B), (C) an 11.7 mm well-circumscribed, hypervascular nodule. (D) Normal right testis.

A 32-year-old male with a past medical history of occasional hematospermia and two previous episodes of orchiepididymitis presented with an enlarged left testis. Ultrasonography of the left testis documented multiple nodular, hypoechogenic, and hypervascularized lesions, the largest measuring 11.7 mm (Figure 1).

**Figure 2:**
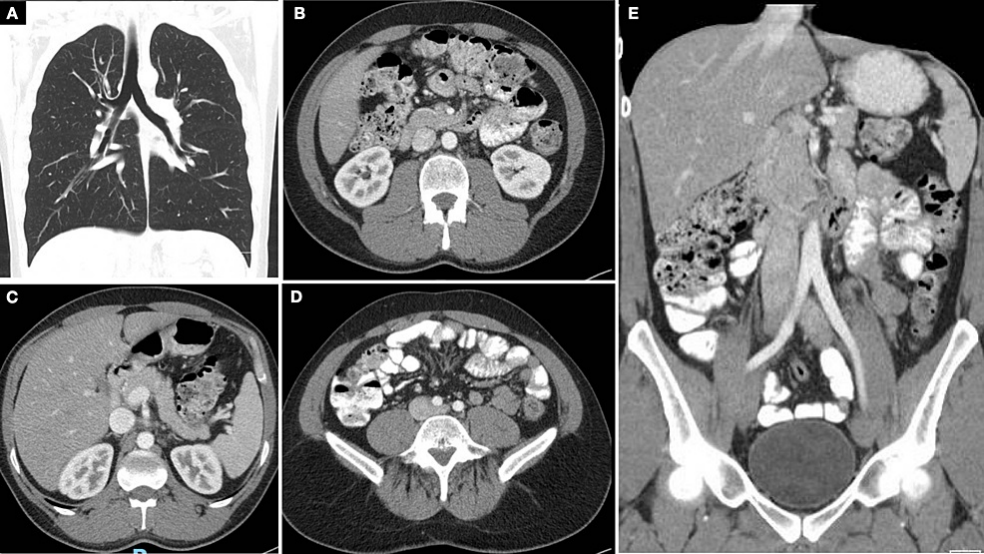
TAP CT scan without evidence of hepatic, pulmonary, and lymph node metastases. (A) Coronal axis view of the lungs. (B), (C), (D) Axial and coronal (E) axis views of the abdomen and pelvis. TAP CT: computer tomography of the thorax, abdomen, and pelvis

Serum tumor markers alpha-fetoprotein (AFP) and beta-human chorionic gonadotropin (Beta-HCG) were normal, and lactate dehydrogenase (LDH) was 198 UI/L (Table 1). A CT scan of the thorax, abdomen, and pelvis (TAP) excluded metastatic disease (Figure 2).

**Table 1:** Laboratory parameters during the disease course AFP: alpha-fetoprotein; Beta-HCG: beta-human chorionic gonadotropin; CPR: C-reactive protein; FSH: follicle-stimulating hormone; LDH: lactate dehydrogenase; LH: luteinizing hormone; MCV: mean corpuscular volume; PSA: prostate-specific antigen, BEP: bleomycin, etoposide, and cisplatin

Parameter	At diagnosis	After orchiectomy	At recurrence	After BEP	Reference values
Hemogram					
Erythrocytes	4.96	5.02	5.07	4.17	4.5-5.6 x10^12^/L
Hemoglobin	15.0	14.8	15.7	13.1	13.7-17.2 mg/dL
MCV	90	94	93	90	83-98 fL
Hematocrit	44.6	47.0	47.3	38.9	50-50%
Leucocytes	8.9	6.4	6.3	5.8	3.7-9.5 x10^9^/L
Neutrophils	68.9 (6.13)	52.5 (3.36)	53.9 (3.42)	65.8 (3.79)	% (1.5-6.5 x10^9^/L)
Platelets	205	248	202	204	170-430 x10^9^/L
Creatinine	1.0	0.79	1.01	0.67	0.7-1.3 mg/dL
LDH	198	204	268	213	85-227 UI/L
AFP	1.8	2.6	2.0	2.0	<8.8 ng/mL
Beta-HCG	<1.0	<1.0	<2.0	<1.0	<2.0 UI/L
PSA			3.17		<4.0 ng/mL
Free testosterone		17.1	9.2		8.8-27.0 pg/mL
Total testosterone		3.49	4.35		2.49-8.36 ng/mL
FSH		15	23.6		1.4-18.1 mUI/mL
LH			7.1		1.5-9.3 mUI/mL
CRP			0.5		<0.6 mg/dL
Urinalysis					
Density			1.028		
pH			6.0		
Proteins			++		0.7-2.0 g/L
Glucose			No		
Ketones Bodies			No		
Leucocytes			+		
Hemoglobin			+++		

The patient underwent a left radical inguinal orchiectomy with the placement of a testicular prosthesis. Histopathology analysis documented a classic, pure seminoma, 30 mm of the major axis, with rete testis invasion but without vascular invasion and free surgical margins (pT1bN0M0 - stage IA). After discussing all adjuvant treatment options, a decision for active surveillance was made with serial tumor markers and imaging.

Two years later, he presented with a two-week duration of urinary retention alternating with pollakiuria, a feeling of incomplete bladder emptying, dyspareunia, and anejaculation. The digital rectal examination showed an enlarged, nodular, and painful prostate. Blood tests documented a normal leucocyte count, CRP 0.5 mg/dL, and serum creatinine 1.01 mg/dL. Urinalysis showed moderate hematuria and a negative bacteriologic exam. Tumor markers were prostate-specific antigen (PSA) 3.17 ng/dL, LDH 268 UI/L and normal AFP, Beta-HCG, follicle-stimulating hormone (FSH), luteinizing hormone (LH), and testosterone (Table 1).

**Figure 3:**
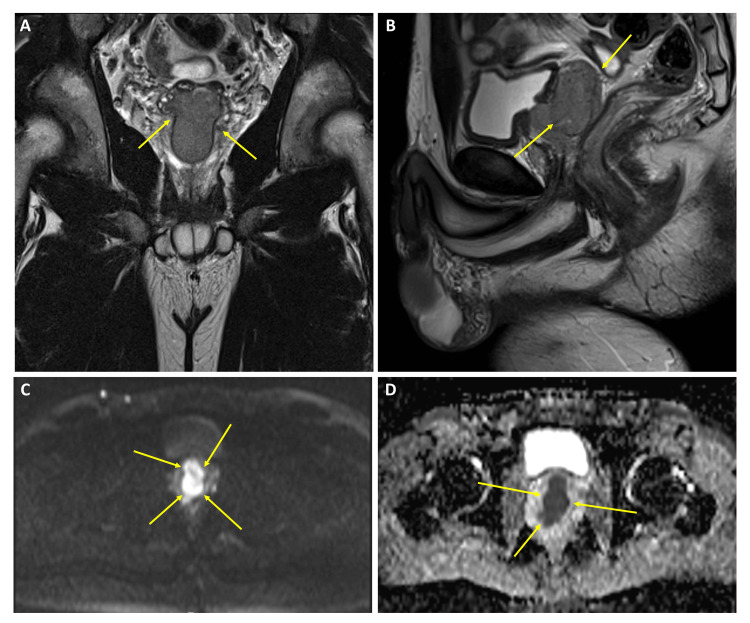
Pelvic MR with a central nodular solid prostatic mass, 40×50×25 mm (arrows), adjacent to the urethra with direct invasion of the right seminal vesicle and no clear cleavage plane with the right anterolateral wall of the rectum. (A) T2, coronal axis; (B) T2, sagittal axis; (C) hyperintense lesion on diffusion restriction; and (D) hypointense lesion on MAPA ADC. MR: magnetic resonance

Pelvic magnetic resonance (MR) documented a central nodular solid prostatic mass, 40x50x25 mm, adjacent to the urethra with direct invasion of the right seminal vesicle and no clear cleavage plane with the right anterolateral wall of the rectum (Figure 3).

**Figure 4:**
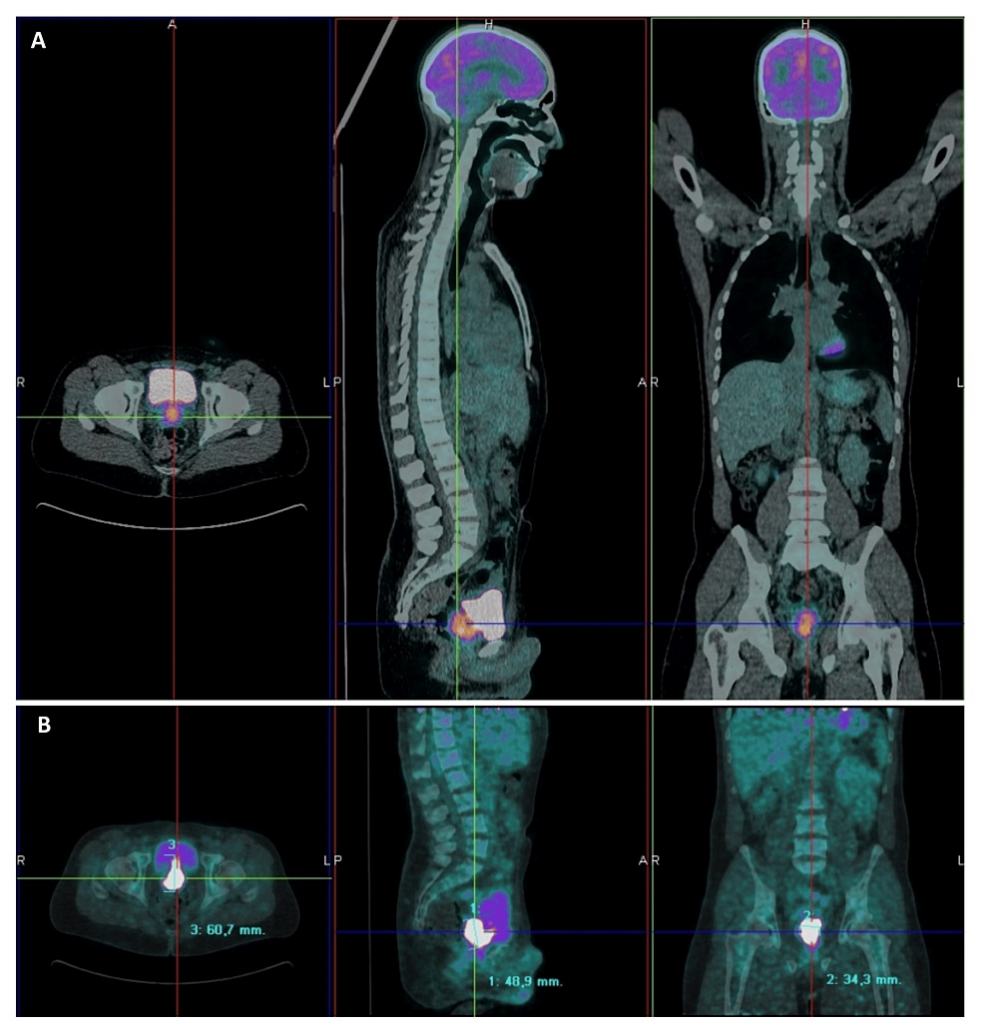
PET-CT with a large focus on uptake involving the anterior rectal wall, posteroinferior bladder wall, and the basis of both seminal vesicles, 49x61x34mm, (A) SUV 18.87 and (B) 21 in later images. PET-CT: positron emission tomography CT; SUV: standardized uptake value

On positron emission tomography CT (PET-CT), the prostatic mass measured 49x61x34mm and had high metabolic activity (standardized uptake value (SUV) 18.87), extending to the anterior rectal wall, posteroinferior bladder wall, and the base of both seminal vesicles, and no distant metastases (Figure 4).

**Figure 5:**
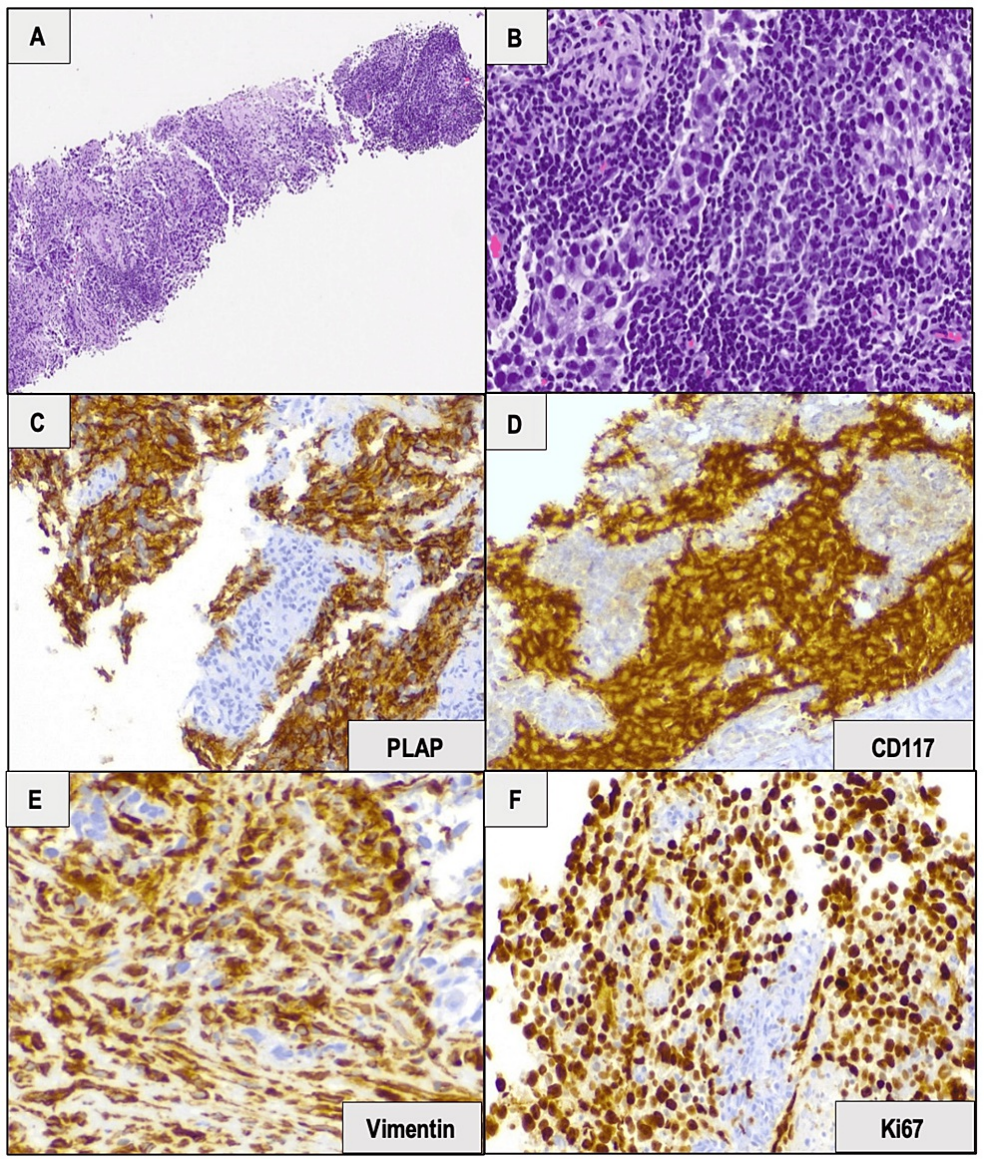
(A) Low power view of TRUS biopsy showing extensive involvement of prostatic parenchyma by tumor cells. (B) High power view revealing the infiltrating tumor cells with a sheet-like growth pattern admixed with small lymphocytes. Tumor cells were large with pale cytoplasm and polygonal nuclei and were diffusely (C) positive with PLAP IHC stain; (D) positive with CD117 IHC marker; (E) positive with vimentin IHC marker; and (F) Ki67 IHC stain depicted a high proliferative index (90%) (x400 each). The tumor cells were also negative for AE1/AE3, PSA, chromogranin, and synaptophysin (not shown). Therefore, it features an immunophenotype consistent with prostatic metastasis of seminoma. IHC: immunohistochemical; TRUS: transrectal ultrasound-guided; PSA: prostate-specific antigen

Transrectal ultrasound-guided (TRUS) biopsy was performed and was consistent with prostatic metastasis of seminoma, as immunohistochemical staining of tumor cells was positive for PLAP, CD117, and vimentin and negative for AE1/AE3, PSA, chromogranin, and synaptophysin. Ki67 IHC stain depicted a high proliferative index (90%) (Figure 5).

**Figure 6:**
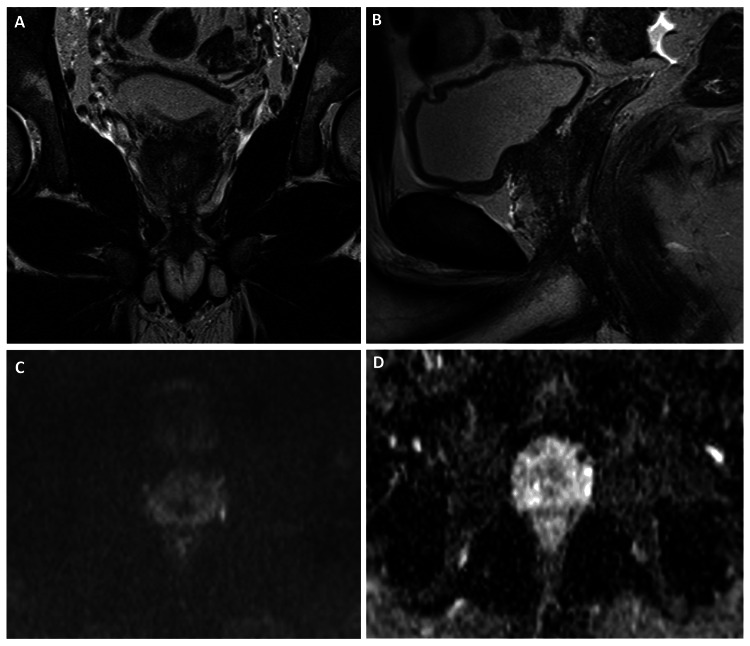
Pelvic MR post-ChT with complete imagiologic response. (A) T2, coronal axis; (B) T2, sagittal axis; (C) hypointense lesion on diffusion restriction; and (D) hyperintense lesion on MAPA ADC. ChT: chemotherapy; MR: magnetic resonance

After a multidisciplinary team discussion, treatment was started with bleomycin, etoposide, and cisplatin for four cycles, with complete resolution of symptoms after the first cycle. Four months later, the PET-CT and pelvic MR documented a complete imagiologic and metabolic response (Figure 6).

TRUS was done to confirm the complete pathologic response. After five years, the patient maintains regular active surveillance with periodic clinical examination, TAP CT, and tumor markers (Beta-HCG, AFP, and LDH), without recurrence of the disease.

## Discussion

Testicular stage I seminomas are the most frequent presentation of GCT in young men, with a cancer-specific survival of more than 94%, regardless of treatment approach. After radical inguinal orchidectomy, most men remain on active surveillance for at least five years without recurrence [4]. Tumors larger than 40 mm and/or with rete testis invasion have a worse prognosis, with relapse rates of up to 35%. Aparicio et al. reported that the presence of any of these risk criteria was related to a higher recurrence rate (p=0.048). Three-year disease-free survival (DFS) was 88.1% for patients on active surveillance (93.5% for cases with no risk factors, 83.7% for patients with tumor size greater than 40 mm, and 78.3% for those with rete testis involvement) and 98.0% for those treated with adjuvant chemotherapy (ChT) (two risk factors) [4,5]. Based on these data and taking into account that seminomas are chemo-sensitive tumors that can be treated with ChT at recurrence with a high probability of cure, adjuvant ChT with one course of carboplatin, dosing to an area under the curve of 7, should be discussed with patients not willing or not able to undergo surveillance or higher-risk patients; the other cases should be enrolled in an active surveillance program and treated at recurrence, as in the present case [1,2].

The metastatic spread of testicular GCTs is thought to occur through the lymphatic system to the retroperitoneal lymph nodes. Visceral metastases, when present, involve most often the lungs, liver, and brain. In the case reported, the patient presented with a painful prostatic mass suggestive of a prostatic abscess, but a neoplastic etiology could not be excluded. Prostate adenocarcinoma, although more common in older men (median age at diagnosis of 68 years), is diagnosed below age 50 in 10% of cases in the USA, doubling the proportion of cases diagnosed in 1995 when PSA screening started being implemented. These cases diagnosed at a younger age have a higher risk of metastatic disease and higher mortality compared with older age groups [6].

With a previous diagnosis of testicular seminoma, young age, and normal serum PSA, after exclusion of prostate infection, prostate metastasis from testicular seminoma becomes the most likely diagnosis. Primary extragonadal GCTs (EGGCTs) are rare tumors with an incidence of 1,8-3,4/million, accounting for 1% to 3% of GCTs. They can occur in almost any midline location, from the brain to the coccyx, being more frequent in the anterior mediastinum, retroperitoneum, and brain [3,7]. Although the etiology is not clearly defined, there are two main hypotheses for the histogenesis of primary EGGCT: the first one postulates that GCTs originate from pluripotent stem cells that can transform into neoplastic germ cells, which may explain the origin of tumors outside the midline, like prostate GCTs. The other hypothesis suggests that germ cells may be sequestered during their migration from the yolk sac to gonadal regions, resulting in midline GCTs [3,7-10].

The diagnosis of EGGCTs is made by histology and has implications for prognosis and therapeutic approach. Due to the rarity of EGGCTs, primary testicular GCT must be excluded, and histological evaluation is required to exclude undifferentiated carcinomas or other cancers. Non-seminoma GCT (NSGCT) accounts for 60% of primary EGGCT in adults and presents in midline locations [3,7]. However, EGGCTs arising from the prostate gland are very rare, with fewer than 20 cases reported (teratoma, seminoma, or other NSGCT subtypes) [10,11]. Regardless of location (with primary tumors of the mediastinum having the worst prognosis), EGGCT seminomas have a better prognosis than NSGCT, with a five-year DFS of 82.3% and a five-year recurrence rate of 6-12%, with recurrence occurring mostly in the first two years, like testicular seminomas [7,10,12,13].

In the past, complete surgical resection of the tumoral masses was the standard therapy for EGGCTs, which was associated with a poorer prognosis, particularly in mediastinal EGGCTs and EGGCTs with NSGCT components. The introduction of cisplatin-based ChT has dramatically improved the prognosis of these patients and made cisplatin-based ChT the treatment of choice for advanced GCTs [3]. Radical surgery is reserved for residual lesions persisting after ChT, and orchiectomy is not recommended in the absence of clinical or ultrasound evidence of a testicular tumor. In the case of localized, resectable EGGCTs (and mostly in the case of teratoma), upfront radical surgery followed by ChT has advantages in DFS when complete surgical excision is possible [3].

The cases of prostate EGGCTs (primary or metastatic) reported in the literature were mainly treated before 2000, when complete surgical resection with pelvic exenteration was the standard of care. More recent cases were treated primarily with cisplatin-based ChT and underwent surgical resection only if residual lesions persisted (as in testicular GCTs), avoiding the unnecessary morbidity of surgery in patients without evidence of residual tumor [10-17]. In the case now reported, a TRUS biopsy established the diagnosis of a prostatic metastasis of a previously diagnosed primary testicular seminoma. The patient was thus treated with four cycles of BEP with a complete radiological and metabolic response. After five years of follow-up, the patient is free of recurrence and avoids the morbidity of any pelvic surgery, documenting the efficacy of the isolated systemic treatment approach.

## Conclusions

GCTs have a good prognosis regardless of primary and stage of diagnosis. Seminomas have a better prognosis than NSGCT, with a high rate of cure and longer survival.

The metastatic spread of testicular GCTs occurs through the lymphatic system to the retroperitoneal lymph nodes. Visceral metastases, when present, involve mostly the lungs, liver, and brain. Cisplatin-based ChT followed by surgery of residual masses is the standard-of-care treatment of advanced disease with a possible cure.
